# Editorial: The mechanisms of degenerative and geriatric disorders of the musculoskeletal system

**DOI:** 10.3389/fmed.2024.1422428

**Published:** 2024-05-24

**Authors:** Lei Kuang

**Affiliations:** Department of Spinal Surgery, The Second Xiangya Hospital of Central South University, Changsha, Hunan, China

**Keywords:** musculoskeletal disorders, 3D printing, sarcopenia, bioinformatics, mechanism

The functional and structural changes of degenerative and geriatric musculoskeletal system include osteoarthritis, osteoporosis, intervertebral disc degeneration, and sarcopenia. In the context of global aging, the incidence of these diseases are increasing rapidly. Despite the significant investment of time and resources into research of their mechanism, the etiology and pathogenesis of these diseases are not fully understood. Therefore, the present treatment of these diseases can only focus on the late-stage manifestations rather than the cause. We are pleased to present this Research Topic on the mechanisms of degenerative and geriatric musculoskeletal disorders, investigating both the basic science and advancements to better understand them.

Currently, bioinformatics is widely employed in research on various diseases. Integrating relevant bioinformatics studies can more accurately screen differential genes, enabling the exploration of potential disease mechanisms. In the article *Revealing the impact of TOX3 on osteoarthritis: insights from bioinformatics*, the authors discovered new diagnostic genes, TOX3, for osteoarthritis (OA) through bioinformatics, which would establish a theoretical foundation for future personalized and accurate treatment of OA patients (Wang et al.). The authors of *Bioinformatic analysis of the molecular mechanisms underlying the progression of bone defects* revealed that three sets of molecular processes served important roles in the progression of bone defects (Liu et al.). These related genes may provide new insights into the treatment of orthopedic diseases.

Recent studies revealed the existence of sarcopenia as a high-risk factor for cirrhosis-related events, but pathophysiological mechanisms of sarcopenia in this disease are multifactorial and complex. The authors of *Plasma pentosidine as a useful biomarker of sarcopenia, low gait speed, and mortality in patients with cirrhosis* were the first to find that plasma pentosidine level was significantly and independently associated with sarcopenia, low gait speed, and mortality (Saeki et al.).

In addition to the investigation of the pathogenesis of degenerative diseases, the exploration of the treatment of the mechanism is also important. *The effect of sequential perioperative intravenous tranexamic acid in reducing postoperative blood loss and hidden blood loss after posterior lumbar interbody fusion: a randomized controlled trial* showed, for the first time, that sequential intravenous application of 1 g of TXA at 24-h intervals 1–3 days after the operation could effectively reduce blood loss without increasing complications, which provides new ideas for bleeding management after spinal fusion (Dong et al.). *Comparison of curative effect between OBS assisted by 3D printing and PFNA in the treatment of AO/OTA type 31-A3 femoral intertrochanteric fractures in elderly patients* also gave us a perspective on innovative treatments on fractures in elderly patients (Huang et al.). *Traditional Chinese exercises on pain and disability in middle-aged and elderly patients with lumbar disc herniation: a systematic review and meta-analysis of randomized controlled trials* suggested that traditional Chinese exercises could be a feasible and safe treatment option for lumbar disc herniation (Zhang et al.).

We anticipate that this Research Topic will provide researchers and clinicians with valuable insight into the challenges that degenerative and geriatric diseases pose, as well as novel strategies to investigate these diseases.

## Author contributions

LK: Writing – review & editing, Writing – original draft.

